# Forward Osmosis Desalination Using Thermoresponsive Ionic Liquids: Bench-Scale Demonstration and Cost Analysis

**DOI:** 10.1021/acs.iecr.4c03784

**Published:** 2025-04-02

**Authors:** Andrew Z. Haddad, Akanksha K. Menon, Ravindra Revanur, Jennifer Klare, Jeffrey J. Urban, Robert Kostecki

**Affiliations:** † Energy Storage & Distributed Resources Division, Lawrence Berkeley National Laboratory, Berkeley, California 94720, United States; ‡ George W. Woodruff School of Mechanical Engineering, Georgia Institute of Technology, Atlanta, Georgia 30332, United States; § Porifera Inc., San Leandro, California 94577, United States; ∥ Molecular Foundry, Lawrence Berkeley National Laboratory, Berkeley, California 94720, United States

## Abstract

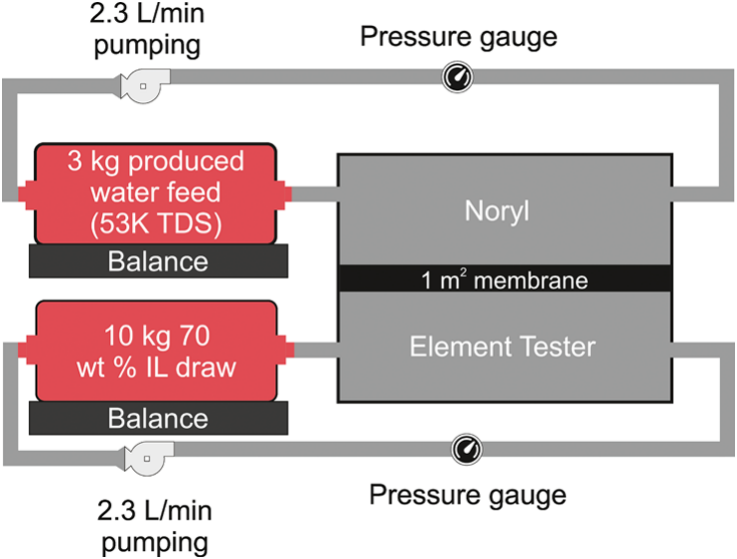

Forward osmosis (FO) desalination using thermoresponsive ionic liquid (IL)–water mixtures is a promising technology for treating nontraditional water sources. However, its demonstration has primarily been at the lab-scale, with water flux and recovery values that are not representative of realistic applications. In this work, the performance of tetrabutyl-phosphonium trifluoroacetate (P_4444_TFA), as well as a new dual draw of P_4444_TFA with tetrabutyl-ammonium trifluoroacetate (N_4444_TFA) is characterized. The dual draw combines the higher osmolality of one IL with the lower critical solution temperature (LCST) of the second IL to outperform its constituents at the same total concentration of IL in water (70 wt %). Experiments were first performed in a lab-scale coupon tester to understand the effects of draw osmotic pressure and viscosity on water flux through the membrane. Bench-scale experiments were then performed in an element tester with a 1 m^2^ membrane area to evaluate the performance of IL-based FO for the desalination of produced water feed from oil and gas. Specifically, 10 kg of IL-water draw solution was used with 3 kg of real produced water feed, resulting in water recoveries of 60% with initial and final water fluxes of 14 LMH and 3 LMH, respectively. The bench-scale experimental results were used as inputs for a cost analysis, yielding a levelized cost of water (LCOW) of $1.18 per m^3^. This reveals the potential of IL-based draw solutions for cost-effective desalination of challenging feedwaters using FO.

## Introduction

Water is a critical resource that is oftentimes the hidden backbone of all economic activity influencing sectors such as agriculture and energy production.^1,2^ With global population growth, the demand for freshwater has increased significantly,^3,4^ prompting the consideration of alternative water sources that are underutilized.^5,6^ The treatment of nontraditional water sources, such as produced waters from oil and gas extraction or industrial discharges (*e.g.*, acid mine drainage), presents unique opportunities to augment freshwater supply. Yet, the utilization of these waters is not without significant challenges, which can be attributed to their high salinities, often greater than 35,000 ppm total dissolved solids (TDS), and complex compositions.^7,8^ Treatment using membrane processes such as reverse osmosis (RO) is often unviable due to the high operating pressures (>75 bar) beyond the stability of commercial polymeric membranes, as well as scaling or fouling at the membrane interface. Consequently, these waters are disposed either through deep well injection^9,10^ or discharged in evaporation ponds rather than being treated for reuse.^1,8^ The potential to turn nontraditional waters into freshwater feedstocks could have immense implications – in the United States alone, produced water from oil and gas extraction accounts for ∼ 2.1 billion barrels (bbl) of wastewater per year.^10^

One promising solution to treat these types of nontraditional waters is forward osmosis (FO). Literature has shown that FO exhibits a higher fouling and scaling resistance relative to RO,^11,12^ making it suitable for variable water compositions with minimal pretreatment and membrane maintenance or replacement.^13,14^ In FO, the osmotic pressure difference between a saline solution (feed) and a higher concentration solution (draw) causes water to spontaneously diffuse through a semipermeable membrane until equilibrium is attained by diluting the draw. However, implementation of FO is limited by the energy needed to regenerate the diluted draw to yield clean water and replenish the concentrated draw for a continuous desalination process. This is because conventional draws (*e.g.*, aqueous NaCl solution) rely on evaporating water using thermal or membrane distillation that is constrained by its large enthalpy of vaporization (liquid–vapor separation).^15−18^ Regeneration can also be achieved by using RO after FO, but this is more energy intensive than direct treatment of that same feed (i.e., RO alone).^19^ Other FO draws are based on volatile species, such that the solute (*e.g.*, NH_3_–CO_2_) is vaporized instead of water to reduce the regeneration energy input.^20^ Despite the high osmotic pressure and water flux obtained using such a thermolytic draw, the high reverse solute flux necessitates further treatment to improve permeate quality, in addition to increasing the draw replacement cost.^21^

**Figure 1:**
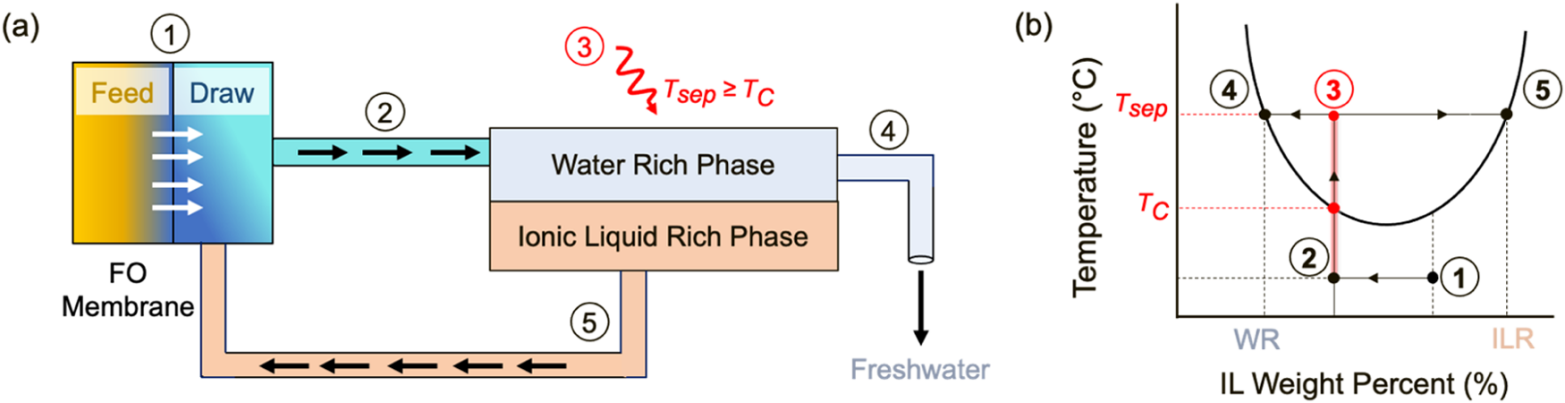
(a) Schematic showing the FO desalination process comprising draw dilution (step 1–2) and thermal regeneration (step 2–3). (b) Illustration of the LCST phase diagram for IL-water mixtures resulting in the formation of a water-rich (W*R*) and IL-rich (ILR) phase above the critical temperature, *T*_*C*_.

Beyond conventional draws, recent literature has demonstrated FO desalination using ionic liquids (IL) that exhibit a lower critical solution temperature (LCST) phase behavior with water.^22−24^ In this process, thermoresponsive ILs draw water across the FO membrane, and the diluted draw is then heated above its critical temperature (*T*_*C*_) to separate into a water-rich (permeate) and an IL-rich (reused as draw) phase as shown in Figure 1. We previously demonstrated that solar-thermal energy can be used to regenerate the draw solution (liquid–liquid separation) given the low separation temperatures and enthalpies required by these ILs.^25^ However, these previous experiments were performed using a lab-scale membrane test cell in which the desalination performance was limited by the low flow rate (cross-flow velocity) and water recovery that are not representative of operating conditions in a real application. Furthermore, the ILs studied were tetrabutylphosphonium dimethylbenzenesulfonate (P_4444_DMBS) and trifluoroacetate (P_4444_TFA), which had low osmotic pressures at concentrations <70 wt % that resulted in low water recoveries. This is because higher osmotic pressures are typically correlated with higher temperatures (*T*_*sep*_) needed to induce phase separation between the IL and water.

To address these limitations, herein we report a new dual draw and demonstrate the bench-scale testing of IL-based FO under realistic operating conditions for the first time. The draw is composed of an aqueous mixture of two ILs, namely tetrabutylphosphonium- and tetrabutylammonium trifluoroacetate (P_4444_TFA and N_4444_TFA), which leverages the (i) higher osmotic pressure of the ammonium-based IL, and (ii) the lower LCST of the phosphonium-based IL, while keeping the anion the same. Desalination experiments were performed in FO membrane modules, first with a lab-scale coupon tester (0.289 cm^2^ membrane area) to understand the effects of draw viscosity and osmotic pressure on the resulting water flux, and then with an element tester (1 m^2^ membrane area) to evaluate the bench-scale performance of this dual draw for desalination of real oil and gas produced water. The bench-scale experimental results are then used as inputs to perform a technoeconomic analysis and assess the viability of FO desalination of nontraditional feeds.

## Materials and Methods

### Synthesis of Ionic Liquids

P_4444_TFA and N_4444_TFA were synthesized using a neutralization reaction.^25^ The starting materials were tetrabutylphosphonium hydroxide (TBPH) or tetrabutylammonium hydroxide (TBAH) and trifluoroacetic acid (TCI America). Both reagents were mixed with water in a 1:1 molar ratio, with a slight excess of trifluoroacetic acid, and stirred at room temperature for 24 h. The solution was then added to a separatory funnel and extracted with dichloromethane (DCM); this step was repeated three times. The organic phase was collected and washed with water and extracted three times, and then transferred to a rotary evaporator to remove DCM from the IL. The resultant IL was dried in a vacuum oven at 90 °C for 48 h to remove residual water. ^1^H NMR was performed to confirm the purity of both ILs: (DMSO, δ /ppm relative to TMS). P_4444_TFA: δ = 0.78–0.98 (3H; a), 1.25–1.51 (4H; b,), 2.03–2.19 (2H, c); N_4444_TFA: δ = 0.78–0.98 (3H; a), 1.20–1.40 (2H; b), 1.5–1.7 (2H, c), 3.05–3.2 (2H, d). This is shown in Figure S1a,b.

### Characterization of Ionic Liquid Solutions

Relevant solution properties of aqueous mixtures of ILs were characterized as a function of concentration.

#### Phase Diagram

To obtain the LCST of the ionic liquids, UV–vis spectroscopy was performed using a Cary 60 UV–vis (Agilent Technologies). Cloud point measurements^26^ were used to identify the temperature at which the solution transmittance drops from 100 to 0%, i.e., the point at which phase separation is initiated.

#### Osmotic Pressure

To evaluate the osmotic strength, osmolality measurements were performed using a Wescor 5600XR vapor pressure osmometer (Thermo Fisher Scientific) at 25 °C. Osmolality was converted to an approximate osmotic pressure, π using π = *m*ρ*RT*, where *m* is the measured osmolality, ρ is the density of the mixture (approximated as the density of water), *R* is the molar gas constant, and *T* is the absolute temperature.

#### Viscosity

The viscosity of aqueous IL solutions was measured using an HR-2 rheometer (TA Instruments) with a parallel plate accessory at a shear rate of 100 Hz. Measurements of 40 wt % P_4444_TFA, 70 wt % N_4444_TFA, and N_4444_TFA/P_4444_TFA mixtures at 25 °C confirmed the Newtonian behavior of these LCST ionic liquids.

### FO Desalination Experimental Setup

FO experiments were carried out using commercially available FO modules from Porifera Inc. (San Leandro, CA). The feed solutions used were real produced water samples obtained from the California Resources Corporation (South Mountain oil field), and the composition is shown in Table S3.^25^

#### Coupon Tester

First, a coupon tester with an active membrane area of 0.289 cm^2^ was used to characterize the performance of the IL draw with the thin film composite membrane. This lab-scale setup (see Figure S2) includes a peristaltic pump (flow rate of 0.45 L/min) that is connected to the membrane coupon cell (specifications in Table S1), with feed and draw containers placed on a scale to measure mass change over time.

#### Element Tester

Bench-scale experiments were performed using an element tester with an active membrane area of 1 m^2^. The element is made from Noryl (a polymer blend of polyphenylene oxide resin with polystyrene) that is compatible with the ILs. For each experiment, 3 kg of feed solution and 10 kg of draw solution were used, which were placed on scales to measure mass change over time. The feed and draw solutions were circulated through the FO element using two pumps (flow rate of ∼2.3 L/min).

For both sets of experiments (coupon tester and element tester), the mass change was converted to a water flux in liters/m^2^/hour (LMH) by measuring the amount of water that diffuses across a unit area of the membrane over time from the feed to draw side. A transmembrane pressure below 5 psi was maintained during all experiments, which ensures that the membrane envelope remains open and maximizes wetting of the viscous draw solution. All FO experiments were conducted at 25 °C and in PRO mode (membrane active layer faces the draw solution) to minimize concentration polarization from the IL draw solutions.^27^

### Technoeconomic Model

The levelized cost of water (LCOW) is an important indicator of the economic viability of a desalination process.^28^ This cost metric is represented as *c*, which is the ratio of the total cost to the total clean water produced per year, as shown in eq 1
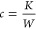
1where *K* is the annual cost of the process encompassing capital expenditures (Capex) and operating expenditures (Opex), and *W* is the clean water produced in one year. Capex considerations include the FO membrane module, ionic liquid synthesis, circulation pumps, post processing (*e.g.*, nanofiltration membrane module), heat exchangers, and balance of plant (BOP) that includes installation, piping, tanks, etc. We note that nanofiltration is used as a post-treatment step to remove residual IL from the water-rich phase, and we have previously shown that this regenerates 100% of the draw and yields high-purity water.^25^

Opex considerations include membrane replacement, electricity for pumping, thermal energy for draw regeneration,^25^ and maintenance costs (estimated to be 10% of overall Opex costs, consistent with desalination analyses in the literature).^29−31^ We note that brine disposal costs are also included in Opex to account for the fact that the FO process has a finite recovery and generates a byproduct that must be managed especially in inland desalination (minimal to zero liquid discharge^28,32^).

The Capex values are summed and annualized by considering the standard capital recovery factor, and then added to the annual Opex. Thus, the annualized cost *K*, can be expressed using eq 2(33)
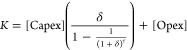
2where a discount rate (δ) of 8% and a lifetime (τ) of 30 years is considered. While economic assumptions are relatively general (the costs of the components do not reflect a specific geographic region), the discount rate is representative of the application of interest. Table S2 lists the technoeconomic model input parameters for FO desalination capacities of 500 m^3^/day (upper limit) and 100 m^3^/day (lower limit) for on-site produced water treatment. Draw regeneration is based on a thermal energy input using a levelized cost of heat (LCOH), while electricity for pumping and nanofiltration is based on a levelized cost of electricity (LCOE).^28,34^

## Results and Discussion

### Solution Properties of Thermoresponsive ILs

**Figure 2:**
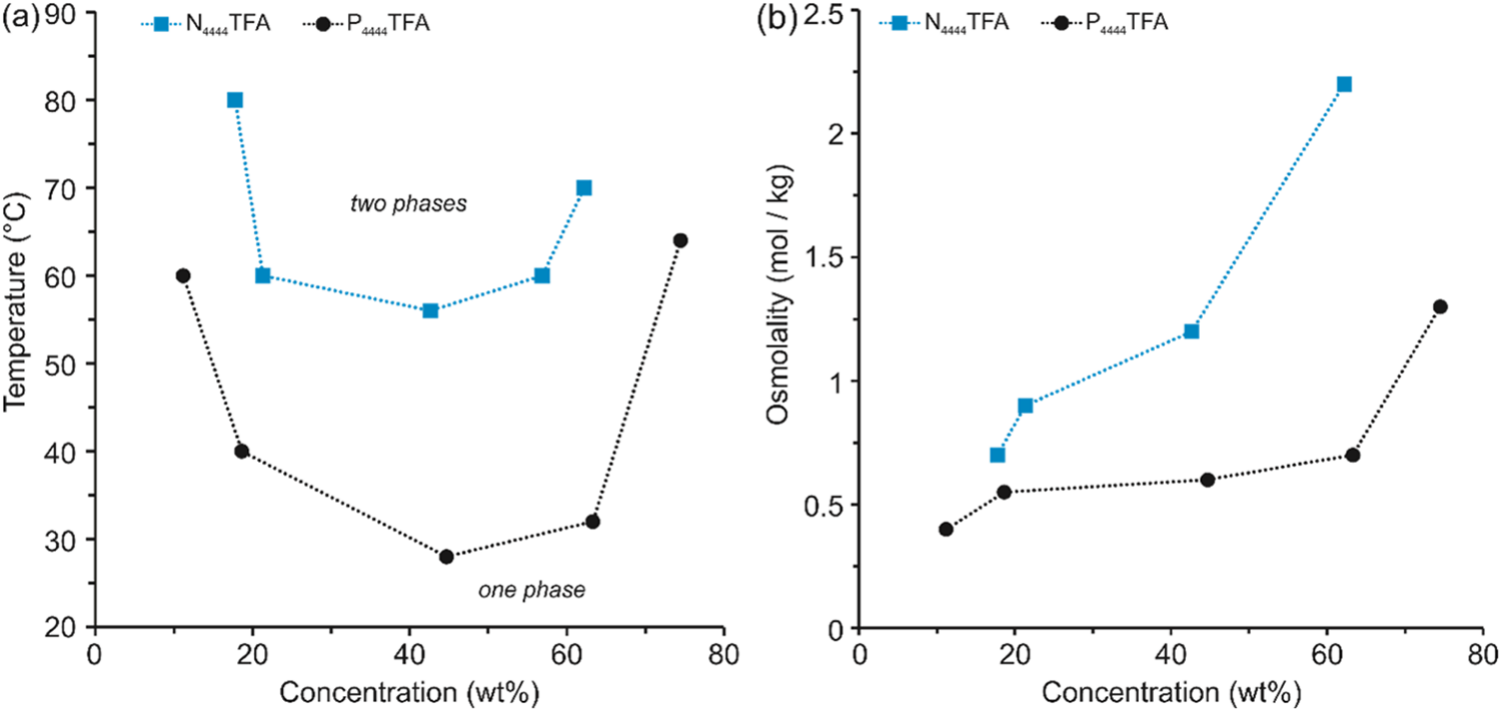
(a) Binary phase diagram of LCST ionic liquids in water, and (b) osmolality data for N_4444_TFA and P_4444_TFA in water at 25 °C. All osmolality measurements have a maximum standard deviation of ± 5% and all cloud point measurements used to construct LCST phase diagrams have a maximum standard deviation of 0.5 °C.

The use of thermally responsive ionic liquids as draw solutions in forward osmosis is impacted by two main criteria, (i) the phase separation temperature needed to form the water-rich and IL-rich phases above the LCST, and (ii) the osmotic pressure of aqueous IL solutions that drives water flux across the membrane. Often, these two characteristics are correlated, meaning that higher osmotic pressures are accompanied by higher LCSTs.^24^ This is confirmed by examining the binary phase diagrams of the ILs used in this study, namely P_4444_TFA and N_4444_TFA in water, as shown in Figure 2.

To benefit from the higher osmolality of N_4444_TFA for higher water recoveries or water fluxes while maintaining the lower LCST of P_4444_TFA for facile draw regeneration, we posit that mixtures of these two ILs can achieve intermediate properties of reasonably high osmotic strength combined with reasonably low LCST. Table 1 shows the experimentally measured values for the osmolality and LCST of the individual ILs at 70 wt % each, as well as their optimal mixture (dual draw) at a total concentration of 70 wt % IL in water, representative of the starting concentration used in FO.

**Table 1:** Solution Properties of P_4444_TFA, N_4444_TFA, and the Dual Draw^1^

draw solution	concentration (wt % IL)	LCST (°C)	osmolality (mmol/kg)	osmotic pressure (bar)	viscosity (cP)^3^
P_4444_TFA/water	70	31	1410	35	14.7
N_4444_TFA/water	70	n/a^2^	2550	63	14.9
N_4444_TFA/P_4444_TFA/water (dual draw)	40/30 (70)	51	2361	58	14.4

1 All draw solutions are in DI water. Osmolality measurements have a maximum standard deviation of ± 5% and all cloud point measurements used to construct LCST phase diagrams have a maximum standard deviation of 0.5 °C.

2 From the phase diagram in Figure 2a, there is no measurable critical temperature for 70 wt % N_4444_TFA.

3 Viscosity data reported at a shear rate of 100 1/s (see Figure S3).

The osmotic pressure data shown in Table 1 would seem to suggest that the 70 wt % N_4444_TFA draw solution will yield the highest water flux. However, no LCST was measured at concentrations above 60 wt %, which precludes this IL from serving as a suitable thermally responsive draw. As a result, no further experiments are performed with N_4444_TFA by itself. The 70 wt % P_4444_TFA has a low LCST of 31 °C, but its osmotic pressure is also low. In comparison, the dual draw of 40 wt % N_4444_TFA and 30 wt % P_4444_TFA (total IL content is 70 wt %) offers some unique advantages: a higher osmotic strength (58 bar) is achieved due to the presence of N_4444_TFA that enables a higher water flux in the FO step, while a moderate phase transition temperature (51 °C) is retained due to the P_4444_TFA content that facilitates regeneration using solar heating. These experimental results validate the hypothesis that mixtures of the phosphonium and ammonium-based TFA ionic liquids are promising. A detailed investigation of IL mixtures across different compositions with insight into the mechanism that leads to such a synergistic performance enhancement has been reported in a separate manuscript.^35^

Apart from the LCST and osmotic strength, viscosity is the other solution property that affects FO performance. Specifically, a high viscosity can impact the water flux adversely during the draw dilution step by inducing concentration polarization, which reduces the osmotic driving force across the membrane.^27,36^ High viscosity also increases pumping energy demands, which contributes to the operating cost. To this end, the viscosity of the individual ILs, as well as their mixture at a total concentration of 70 wt % in water were measured at 25 °C. As shown in Table 1, all 70 wt % draw solutions have similar viscosity values ∼15 cP and show Newtonian behavior (Figure S3). The dual draw exhibits a slightly lower viscosity than its constituent ILs, which again makes it promising for FO. The importance of viscosity and consequently cross-flow velocity on water flux across the membrane is discussed in the following section.

### FO Desalination in a Coupon Tester (Lab-Scale Performance)

**Figure 3:**
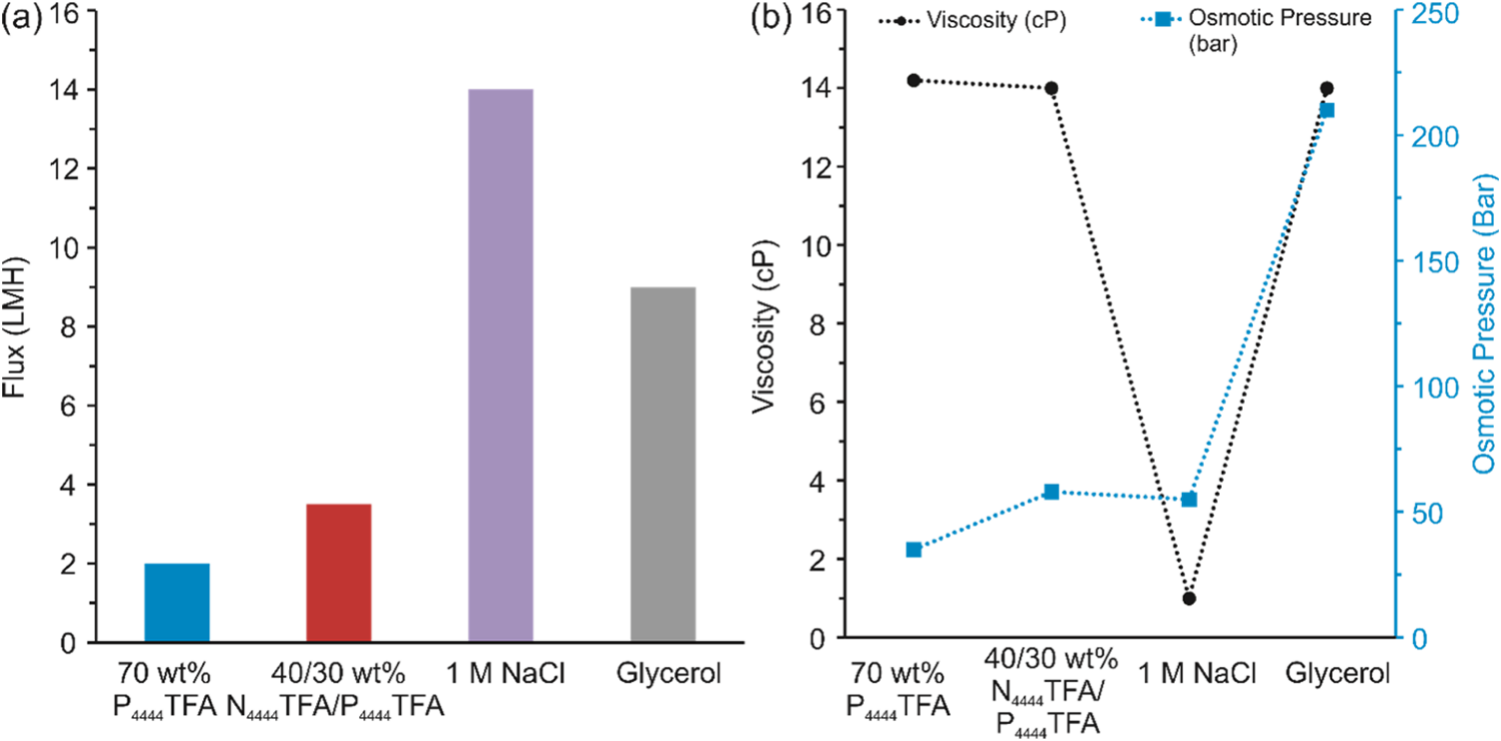
(a) Coupon tester results showing water fluxes obtained using different draws: 70 wt % P_4444_TFA, 40/30 wt % N_4444_TFA/P_4444_TFA, 1 M NaCl, and 70 wt % glycerol. The feed in all cases is produced water. (b) Viscosity (black) and osmotic pressure (blue) of 70 wt % P_4444_TFA, 40/30 wt % N_4444_TFA/P_4444_TFA, 1 M NaCl, and 70 wt % glycerol. All measurements are reported at 25 °C.

FO desalination experiments were first performed in the coupon tester (see Materials and Methods) to evaluate the impact of draw viscosity and osmotic pressure on the water flux. Experiments were performed at an average crossflow velocity of 14.5 cm/s using real produced water feeds from the South Mountain oil field in Southern California (composition shown in Table S3). The water flux values obtained using the thermally responsive IL draws (70 wt % P_4444_TFA and 40 wt % N_4444_TFA/30 wt % P_4444_TFA) are shown in Figure 3a. Specifically, a flux of 2 LMH is achieved by P_4444_TFA, whereas the dual draw achieves a flux of 3.5 LMH – this represents a 75% increase over P_4444_TFA at the same total concentration of IL in water (70 wt %). We note that 70 wt % N_4444_TFA was not tested in FO due to its inability to phase separate at this concentration (see Figure 2a and Table 1).

To better understand the measured flux values, the effect of draw viscosity on crossflow velocity must also be considered as osmotic strength alone does not dictate FO performance.^25^ To this end, two pseudo draw solutions were prepared – the first was a 1 M NaCl solution with a similar osmotic pressure as the dual draw (∼50 bar) but lower viscosity, while the second was a 70 wt % glycerol solution with a viscosity similar to the dual draw (∼14 cP at 25 °C) but higher osmotic pressure, as shown in Figure 3b. A series of FO experiments were performed in the coupon tester using the different draws and real produced water feed. Interestingly, the 1 M NaCl draw achieves a much higher flux of ∼14 LMH compared to only ∼3 LMH with the dual IL draw despite their similar osmotic pressures. Furthermore, the 70 wt % glycerol draw that has a viscosity similar to the dual IL draw, but a significantly higher osmotic pressure (∼200 bar), achieves a flux of only 9 LMH. Collectively, these results reveal that viscosity is the solution property that limits flux, with a high viscosity reducing mixing at the membrane interface (low crossflow velocity) and thereby exacerbating concentration polarization. We note that these pseudo draws are not thermally responsive and are only used to demonstrate that the flux is impacted by a multitude of factors including viscosity and osmotic pressure. The practical use of NaCl as a draw for FO is limited by its energy intensive regeneration (requires evaporation/distillation or RO post FO).^25^ Similarly, separating glycerol from water requires the use of distillation. This highlights the advantage of LCST ionic liquids, as they demonstrate a low energy regeneration process (liquid–liquid phase separation) to yield clean water.

All FO experiments were conducted in PRO mode in order to minimize concentration polarization. However, as shown in Figure 3 it still plays a role depending on the properties of the draw solution. Therefore, enhancing mixing at the membrane interface by increasing the crossflow velocity of the draw solutions can enhance flux, as discussed in the next section.

### FO Desalination in an Element Tester (Bench-Scale Performance)

**Figure 4:**
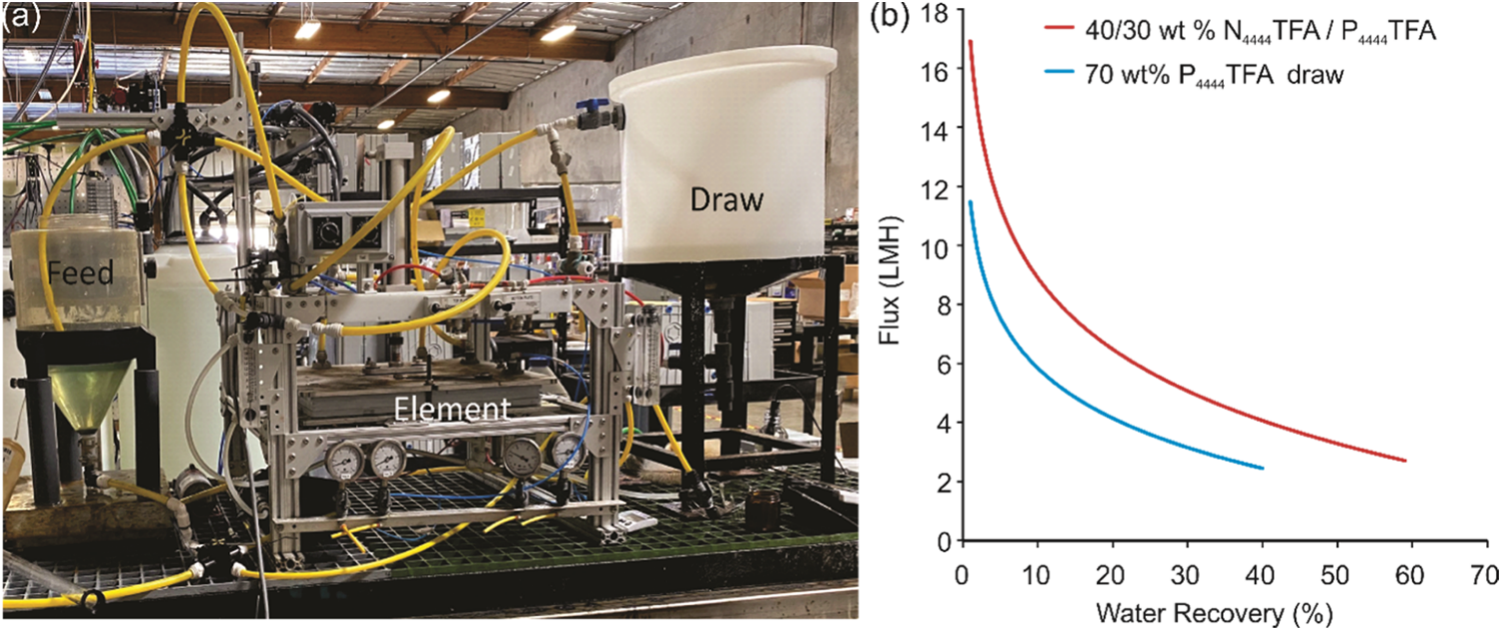
(a) Experimental setup showing the FO element tester (active membrane area of 1 m^2^) using produced water feed and two thermally responsive IL-based draw solutions. (b) Flux vs water recovery using the 70 wt % P_4444_TFA draw (blue curve), and the 40/30 wt % N_4444_TFA/P_4444_TFA dual draw (red curve).

To achieve higher crossflow velocities and enhance the water flux, FO experiments were performed using an element tester (see Materials and Methods), as shown in Figure 4a. The element tester comprises a large 1 m^2^ membrane area coupled with higher capacity pumps that can circulate and uniformly distribute the viscous IL draw solutions, in addition to matching the flow rates of the feed and draw solutions if required. This operational mode enables higher crossflow velocities that are more representative of a real FO desalination system with stacked membrane modules for larger scale deployment. The element tests were performed with both the thermally responsive IL draw solutions, namely 70 wt % P_4444_TFA and 40/30 wt % N_4444_TFA/P_4444_TFA. All tests used produced water feed (Table S3), and were operated at a flow rate that is 10 times higher than our previous lab-scale system.^25^

The water flux as a function of recovery is shown in Figure 4b – as expected, there is a trend of decreasing water flux with increased water recovery. This is attributed to concentration polarization on the feed solution side and dilution of the draw solution over the duration of the experiment. For the 70 wt % P_4444_TFA draw, the initial flux values exceed 10 LMH, a substantial improvement over results from the coupon tester shown in Figure 3a for the same feed and draw solutions – this confirms that increased flow rate and resultant crossflow velocity promote better contact of the draw solution at the membrane interface. As a water recovery of 40% is approached over a duration of 3 h, the water flux reduces to ∼2 LMH. This is confirmed with ionic conductivity measurements of the feed solution before and after FO experiments, yielding values of 36 mS/cm and 60 mS/cm that are in good agreement with 40% concentration of the feed.

To achieve higher fluxes and increase the water recovery further, we used the dual draw at an initial total IL concentration of 70 wt % (40 wt % N_4444_TFA/30 wt % P_4444_TFA). In this case, the initial flux achieved is 17 LMH, owing to the higher osmotic pressure driving force as well as the reduced viscosity of the dual draw compared to P_4444_TFA alone. Correspondingly, a higher water recovery of 60% is achieved in under 3 h and the water flux reduces to 3 LMH, as shown in Figure 4b. This confirms that the viscosity of the IL-based draw solutions, traditionally thought to limit FO performance, can be overcome by using higher crossflow velocities in larger commercial-scale membrane modules. Moreover, a water recovery of 60% demonstrated herein with the dual IL draw and the product water quality (post thermal regeneration and nanofiltration, Table S3) confirms that FO is viable as a membrane desalination process for treating produced water.

### Levelized Cost of Water (LCOW)

**Figure 5:**
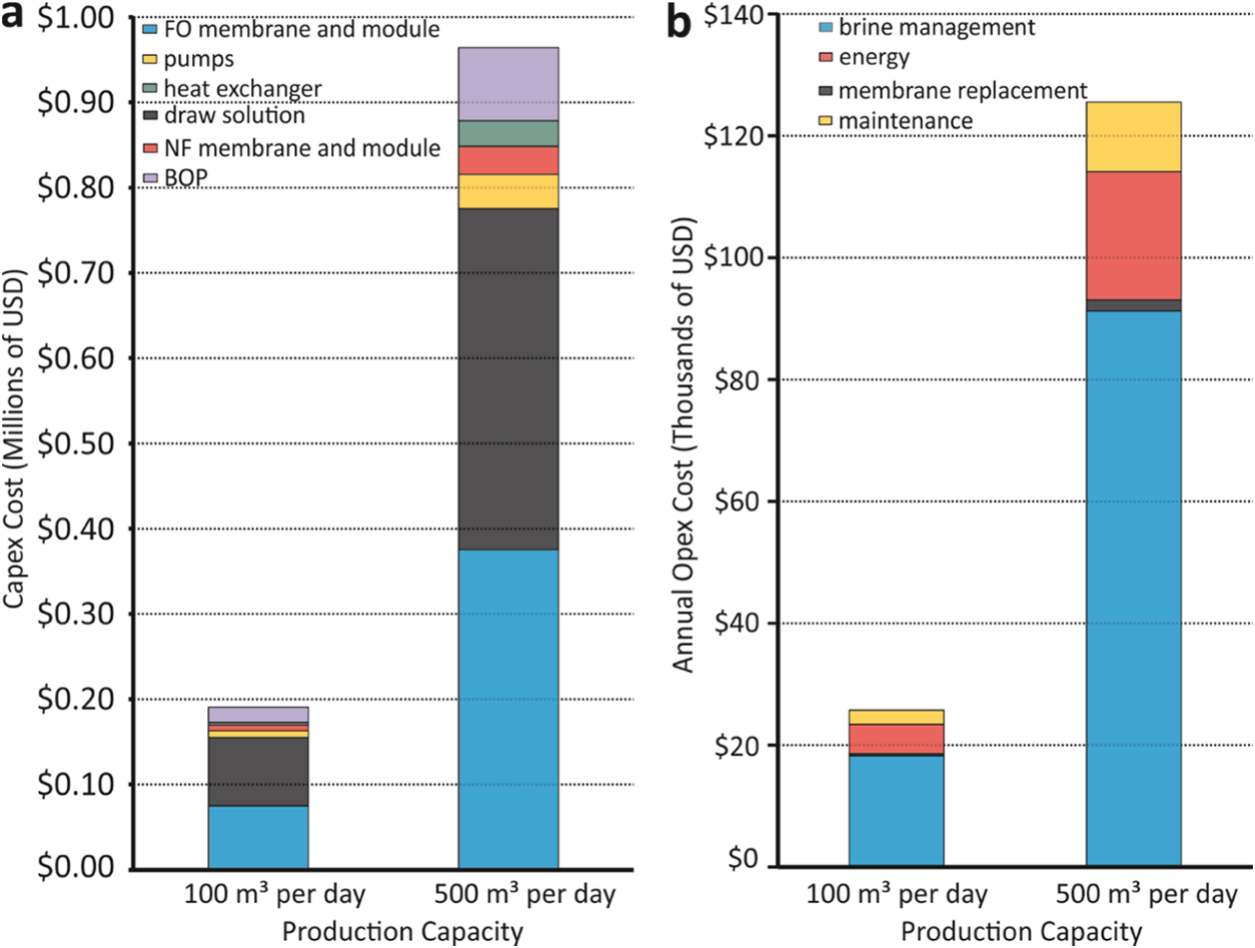
Levelized cost of water (LCOW) calculations for IL-FO desalination at 100 and 500 m^3^ per day production capacities: (a) Capex with component-level contributions, and (b) annual Opex with component-level contributions.

Results from these bench-scale experiments are used as inputs for the technoeconomic modeling and evaluation of the cost of IL-based FO for desalination of nontraditional water sources at pilot scales of 100 m^3^/day and 500 m^3^/day. The total capital expenditure is shown in Figure 5a, which includes component-specific contributions – we find that Capex is dominated by the IL draw solution synthesis (40% of Capex) and the FO membrane modules (42%). Balance of plant constitutes ∼9%, while additional cost contributions from heat exchangers for thermal regeneration and the nanofiltration membrane modules for post-treatment are minimal. The operating expenditure is dominated by brine management and energy costs, each contributing to ∼70 and 18% of Opex, while membrane replacement and maintenance costs are minimal as shown in Figure 5b. We note that the total energy costs account for pumping energy (electricity), energy for draw regeneration (heat), and miscellaneous energy consumption (estimated at 10 kWh per day). After annualizing the Capex over 30 years at 8% and then adding the Opex, an LCOW of $1.18/m^3^ for the dual draw is calculated using eqs 1 and 2.

To interpret the economic feasibility of IL-FO within the desalination market, we compare it with reported LCOW values for different desalination technologies, including FO, RO, multieffect distillation (MED), and mechanical vapor compression (MVC) for treating feeds of similar salinity (∼ 35,000 ppm). FO based desalination using similar thermoresponsive polymer draws^37^ has a reported LCOW value of $2.73/m^3^ for the treatment of seawater utilizing evacuated tube solar collectors, photovoltaics (PV), and waste heat. MVC and MED based seawater desalination are both considerably higher cost, with values ranging from $5.06 to $8.47/m^3^, respectively.^38^ RO is the state-of-the-art technology for seawater desalination, with the LCOW ranging between $0.49 and $0.7/m^3^,^39,40^ depending on recovery rates and system capacity. When paired with renewable energy sources, the LCOW for seawater RO is higher (>$1/m^3^).^41^ There are only a few LCOW analyses for oil and gas produced water feeds. A pilot-scale RO system that treated produced water was projected to achieve LCOW values of $0.92/m^3^ for a full-scale plant.^42^ However, it was noted that extensive chemical pretreatment was necessary to ensure operation. Considering the minimal pretreatment needed for FO desalination, the LCOW of $1.18/m^3^ using thermoresponsive IL draws presented in this work represents a cost-competitive solution, particularly for high-salinity produced waters. Compared to conventional thermal desalination technologies, the energy required for IL-water draw separation is not constrained by the large enthalpy of water vaporization as the LCST enthalpy is orders of magnitude lower (∼10 J/g).^25^ This allows for efficient recovery and reuse of the draw solution, shifting costs from operational expenditures to a fixed capital cost, which reduces the levelized cost over the lifetime of the plant. The results obtained in this work are consistent with the performance model and cost optimization of solar desalination using IL-FO with thermal energy storage in different locations in the United States.^43^

## Conclusions

This work reported the characterization of a thermoresponsive dual IL draw and its bench-scale performance testing using real produced water feeds. The dual draw of P_4444_TFA and N_4444_TFA was shown to combine the higher osmotic strength of one IL with the lower LCST of the second IL. Experiments performed in a lab-scale coupon tester (∼0.3 cm^2^) indicate that both viscosity and osmotic pressure of the draw solution can have substantial impacts on the observed water flux and water recovery in small-scale systems. Bench-scale experiments were performed in an element tester that is representative of larger commercial-scale FO membrane modules (1 m^2^), with higher flow rates being able to overcome limitations traditionally associated with high viscosity draws through increasing crossflow velocity. This in turn results in high water fluxes exceeding 16 LMH and a water recovery of 60% from 3 kg of produced water feed. Finally, these bench-scale results were used to perform an economic assessment which suggests that engineered solar-thermal FO desalination can achieve a levelized cost of water of $1.18/m^3^, making it a cost-competitive option for the desalination of nontraditional feed sources and brines beyond seawater.

## Supporting Information Available

Table showing parameters used for coupon tester experiments and technoeconomic analysis; NMR spectrum of the ionic liquids; plots showing viscosity vs shear rate and mass change over time during FO; table showing the composition of produced water and product water after desalination (PDF)
