# Supplementary material for: Forward Osmosis Desalination Using Thermoresponsive Ionic Liquids: Bench-Scale Demonstration and Cost Analysis
Source: Ind Eng Chem Res. 2025 Apr 2;64(15):7810–7. doi: 10.1021/acs.iecr.4c03784 (PMC12006984; doi:10.1021/acs.iecr.4c03784)
Supplement: Supplementary file 1 — ie4c03784_si_001.pdf [file ie4c03784_si_001.pdf]

Supporting Information

Forward Osmosis Desalination using Thermo-Responsive Ionic Liquids: Bench-scale Demonstration and Cost Analysis

Andrew Z. Haddad[1, $*], Akanksha K. Menon[1,2, $*], Ravindra Revanur[3], Jennifer Klare[3], Jeffrey J. Urban[4], Robert Kostecki[1]

[1] Energy Storage & Distributed Resources Division, Lawrence Berkeley National Laboratory, Berkeley, CA 94720, USA
[2] George W. Woodruff School of Mechanical Engineering, Georgia Institute of Technology, Atlanta, GA 30332, USA
[3] Porifera Inc., San Leandro, CA, 94577, USA
[4] Molecular Foundry, Lawrence Berkeley National Laboratory, Berkeley, CA 94720, USA

[$] Denotes equal contribution.
[*] Corresponding authors: azhaddad@lbl.gov; akanksha.menon@me.gatech.edu

Table S1: Porifera's FO coupon tester parameters

**Parameter**	**Value**
Average Width	3.556 cm
Average Depth	0.081cm
Flow Rate	0.45 L/min
Cross Sectional Area	0.289 cm^2
Cross Flow Velocity	25.77 cm/sec
Shape	Circular
Membrane diameter	4 cm
Flux values reported at temperature of	25° C

Table S2: Input parameters used for LCOW assessment of forward osmosis using ionic liquids

**Parameter**	**Units**	**Value**	**Reference**
Feed flow rate	L/h	• For 500 m^3/day: 20833<br>• For 100 m^3/day: 4166	Calculated based on the clean water demand needed assuming an operation time of 24 hours per day

FO membrane area	m^2	• For 500 m^3/day: 1302<br>• For 100 m^3/day: 260	Calculated based on the experimental water flux of 16 LMH obtained in this work
Number of FO elements		• For 500 m^3/day: 186<br>• For 100 m^3/day: 37	Calculated based on the membrane area of a single FO element (7 m^2) from Porifera Inc.
FO element cost (membrane included)	$/element	2019	Vendor quote (Porifera Inc.)
IL draw solution synthesis cost	$/kg	80	Vendor quote (ProIonic Inc.) for P_{4444}TFA and N_{4444}TFA
Electrical energy cost	$/kWh$_e$	0.2	EIA, 2023 (California industrial electricity price)[1]
Energy needed for draw regeneration	KWh$_{th}$/m^3	35	Based on experimental data from literature[2]
NF energy consumption	kWh$_e$/m^3	0.3	Based on experimental data from literature[2]
NF permeate flow rate	L/h	• For 500 m^3/day: 20833<br>• For 100 m^3/day: 4200	Vendor quote: (VWS Advantage) FilmTec NF270 4040 WET
NF flux (at 12 bar)	LMH	36	Based on experimental data from lierature[2]
NF membrane area	m^2	• For 500 m^3/day: 579<br>• For 100 m^3/day: 117	Calculated based on the NF flux (row above)
Number of NF elements needed		• For 500m^3/day: 15<br>• For 100 m^3/day: 76	Calculated with an element membrane area of 7.6 m^2/element from vendor quote (VWS Advantage) FilmTec NF270 4040 WET)
NF element cost	$/element	433.50	Vendor quote: (VWS Advantage) FilmTec NF270 4040 WET
Heat exchanger cost	$/heat exchanger	2010	Vendor quote (Alfa Laval)
Pump cost	$/pump	1000	Vendor quote (GRUNDFOS)
Pump energy consumption	kWh$_e$/day	5.76	Vendor quote (GRUNDFOS)
Membrane replacement cost	$/$m^3$	0.01	Based on literature[3]
Brine generation rate	% per m^3 water produced	0.4	Calculated based on the experimental water recovery of 60% obtained in this work
Brine disposal costs (minimal liquid discharge)	$/$m^3$	1.25	Based on technoeconomic analysis of minimal-zero liquid discharge[4, 5]

Balance of Plant (BOP): includes installation, piping, tubing, tanks, etc.	%	8	Considered reflective of FO cost analysis at the pilot-scale[6]

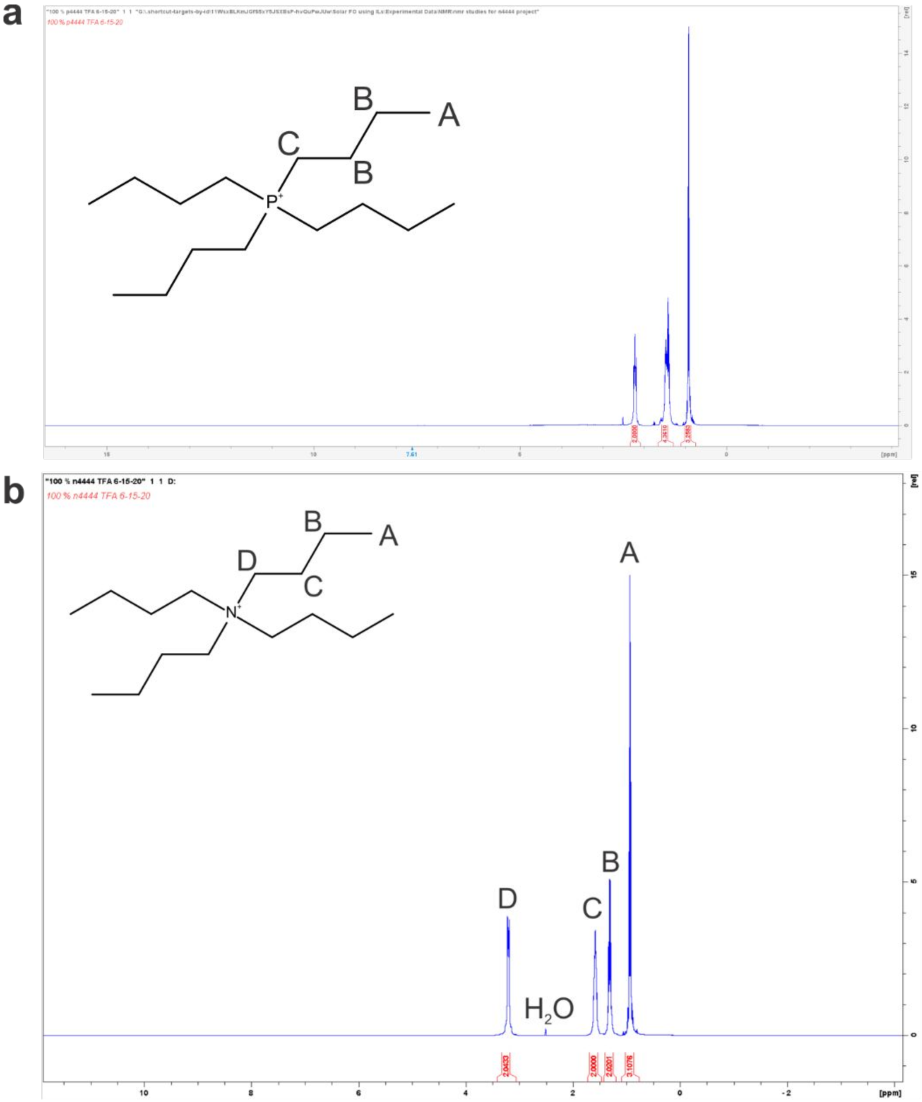

Fig. S1. (**a**) ^{1}H NMR spectrum of P_{4444}TFA and (**b**) N_{4444}TFA

^{1}H-NMR (DMSO, δ /ppm relative to TMS): P_{4444}TFA: δ = 0.78-0.98 (3H; a), 1.25-1.51 (4H; b,), 2.03-2.19 (2H, c). N_{4444}TFA: (DMSO, δ /ppm relative to TMS): δ = 0.78-0.98 (3H; a), 1.20-1.40 (2H; b), 1.5-1.7 (2H, c), 3.05-3.2 (2H, d).

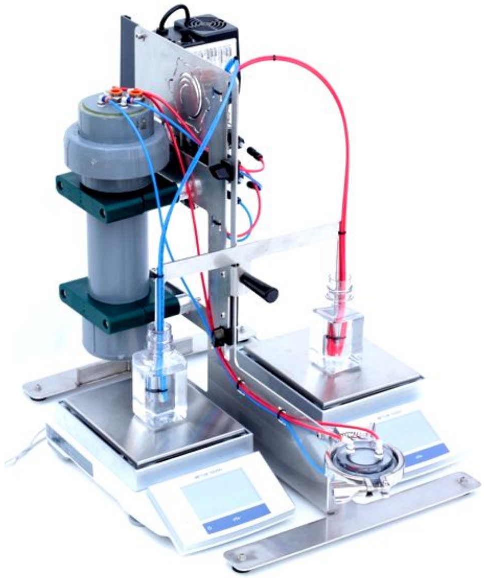

Fig. S2. Porifera FO coupon tester with an active membrane area of 0.289 cm^2 used for lab-scale tests in this work.

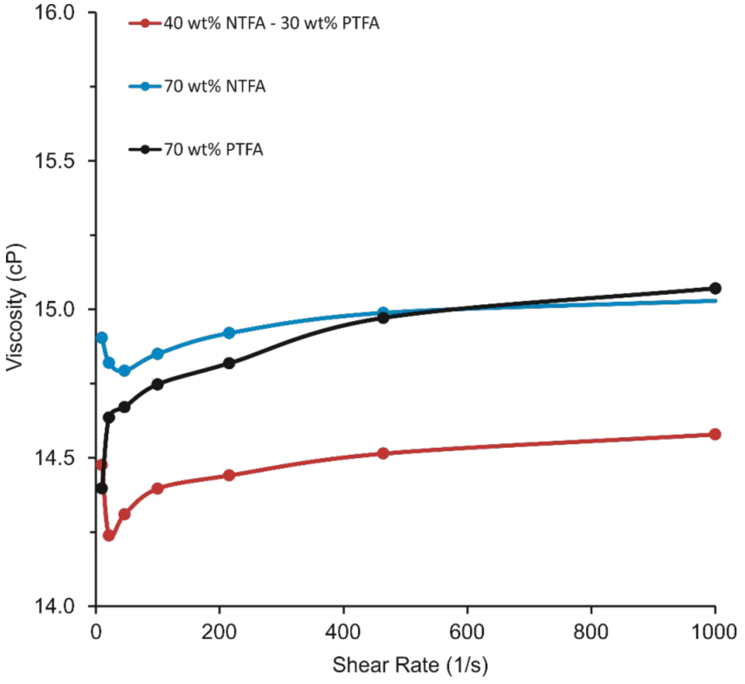

Fig. S3. Viscosity vs. shear rate of the dual draw of 40/30 wt% N_{4444}TFA / P_{4444}TFA (red curve), 70 wt% N_{4444}TFA (blue curve), and 70 wt% P_{4444}TFA (black curve).

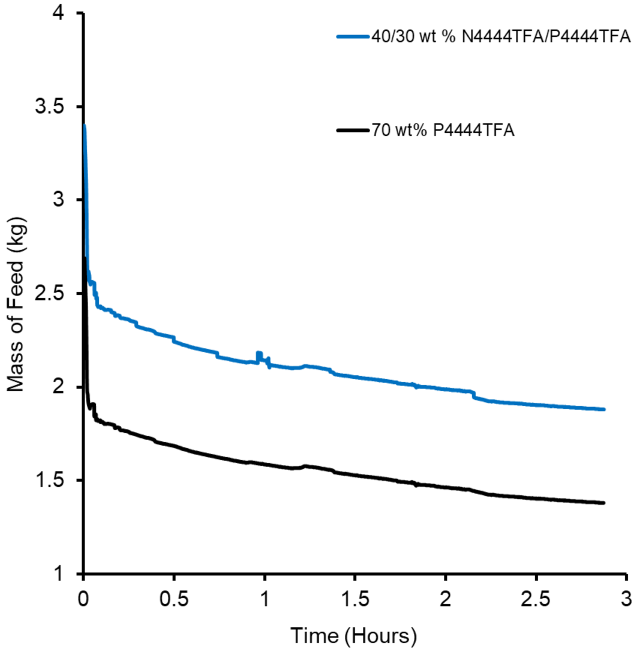

Fig. S4. Mass change (kg) of feed solution vs. time during FO in the element tester with an active membrane area of 1 m^2.

Table S3. Composition of produced water obtained from the California Resources Corporation and used as feed for FO treatment. The product water composition is post-FO dilution and draw regeneration, followed by NF treatment. The analysis was conducted using a third-party EPA certified laboratory following EPA certified sample preparation, digestion, and analysis procedures.

Analyte	South Mountain (Feed)	Product Water
pH	7.09	6.56
Conductivity @ 25 °C	50.8 mS cm^{-1}	2 µS cm^{-1}
TDS @ 180 °C	53,000 mg L^{-1}	264 mg L^{-1}
Total suspended solids	29 mg L^{-1}	0 mg L^{-1}
**Volatile Organics (µg L^{-1})**		
Benzene	3300	ND (RL=500)
sec-butylbenzene	ND	ND (RL=500)
n-butylbenzene	ND	ND (RL=500)
ethylbenzene	360	ND (RL=500)
isopropylbenzne	19	ND (RL=500)
p-isopropylbenzene	ND	-
napthalene	110	ND (RL=2000)
n-propylbenzene	29	-
toluene	3400	ND (RL=500)
1.2.4-trimethylbenzene	94	ND (RL=500)
1,3,5-trimethylbenzene	18	ND (RL=500)
total xylenes	1100	ND (RL=500)

p- an m-xylenes	690	ND (RL=500)
o-xylene	450	ND (RL=500)
gasoline range organics (C4-C12)	29000	ND (RL=50000)
diesel	3500	ND (RL=1000)
Crude oil	ND	ND (RL=3000)
hydraulic oil/motor oil	9400	ND (RL=3000)
oil and grease	17000	ND (RL=3000)
	**Water Analysis (mg L^{-1})**	
hydrogen sulfide	ND	ND (RL= 100)
calcium	5300	ND (RL=100)
magnesium	86	ND (RL=100)
sodium	7700	ND (RL=500)
potassium	43	ND (RL=500)
bicarbonate	92	ND (RL=5000)
bromide	80	ND (RL=100)
Fluoride	ND	ND (RL=200)
chloride	22000	ND (RL=100)
sulfate	27	ND (RL=1000)
ammonia	4	ND (RL100)
ammonium	3.8	ND (RL=100)
nitrite	ND	-
o-phosphate	0.07	ND (RL=20)
total phosphate	1.5	ND (RL=60)
acetic acid	88	-
sulfide	ND	ND (RL=0.1)
lactic acid	61	-
butryic acid	ND	-
propinoic acid	26	-
non-volatile organic carbon	6.1	3.8 (RL=1000)
aluminum	ND	ND (RL=50000)
	**Metal Analysis (µg L-1)**	
antimony	ND	ND (RL=40000)
arsenic	ND	ND (RL=10000)
chromium (vi)	ND	ND (RL=20000)
barium	37000	ND (RL=10000)
beryllium	ND	ND (RL=1000)
boron	16000	ND (RL=50000)
iron	1300	ND (RL=20000)
lithium	810	ND (RL=10000)
manganese	4200	ND (RL=10000)
molybdenum	ND	ND (RL=10000)
mercury	0.065	-
nickel	ND	ND (RL=10000)

selenium	530	ND (RL=30000)
strontium	160000	ND (RL=10000)
zinc	ND	ND (RL=50000)
silica	3300	ND (RL=10000)

ND: not detected; RL = reporting limit (represents the minimum detection limit of the analysis method and/or instrument). Components that were not tested for are marked with a dash or hyphen (-).

References

1. EIA, U., Electric power monthly. *US Energy Information Administration. https://www. eia. gov/electricity/monthly/..*
2. Haddad, A. Z.; Menon, A. K.; Kang, H.; Urban, J. J.; Prasher, R. S.; Kostecki, R., Solar Desalination Using Thermally Responsive Ionic Liquids Regenerated with a Photonic Heater. *Environmental Science & Technology* **2021,** *55* (5), 3260-3269.
3. Samhaber, W. M. N., M. T. Beilstein, Applicability and costs of nanofiltration in combination with photocatalysis for the treatment of dye house effluents. *Journal of Nanotechnology* **2014,** *5*, 476-484.
4. Panagopoulos, A., Techno-economic assessment of minimal liquid discharge (MLD) treatment systems for saline wastewater (brine) management and treatment. *Process Safety and Environmental Protection* **2021,** *146*, 656-669.
5. Menon, A. K.; Jia, M.; Kaur, S.; Dames, C.; Prasher, R. S., Distributed desalination using solar energy: A technoeconomic framework to decarbonize nontraditional water treatment. *iScience* **2023,** *26* (2), 105966.
6. Costa, A. R.; de Pinho, M. N., Performance and cost estimation of nanofiltration for surface water treatment in drinking water production. *Desalination* **2006,** *196* (1), 55-65.
